# Metabolism and Fatty Acid Profile in Fat and Lean Rainbow Trout Lines Fed with Vegetable Oil: Effect of Carbohydrates

**DOI:** 10.1371/journal.pone.0076570

**Published:** 2013-10-04

**Authors:** Biju Sam Kamalam, Françoise Médale, Laurence Larroquet, Geneviève Corraze, Stephane Panserat

**Affiliations:** UR1067 Nutrition Metabolism Aquaculture, Institut National de la Recherche Agronomique, Saint-Pée-sur-Nivelle, France; Institut National de la Recherche Agronomique (INRA), France

## Abstract

The present study investigated the effect of dietary carbohydrates on metabolism, with special focus on fatty acid bioconversion and flesh lipid composition in two rainbow trout lines divergently selected for muscle lipid content and fed with vegetable oils. These lines were chosen based on previously demonstrated potential differences in LC-PUFA synthesis and carbohydrate utilization. Applying a factorial study design, juvenile trout from the lean (L) and the fat (F) line were fed vegetable oil based diets with or without gelatinised starch (17.1%) for 12 weeks. Blood, liver, muscle, intestine and adipose tissue were sampled after the last meal. Feed intake and growth was higher in the L line than the F line, irrespective of the diet. Moderate postprandial hyperglycemia, strong induction of hepatic glucokinase and repressed glucose-6-phosphatase transcripts confirmed the metabolic response of both lines to carbohydrate intake. Further at the transcriptional level, dietary carbohydrate in the presence of n-3 LC-PUFA deficient vegetable oils enhanced intestinal chylomicron assembly, disturbed hepatic lipid metabolism and importantly elicited a higher response of key desaturase and elongase enzymes in the liver and intestine that endorsed our hypothesis. PPARγ was identified as the factor mediating this dietary regulation of fatty acid bioconversion enzymes in the liver. However, these molecular changes were not sufficient to modify the fatty acid composition of muscle or liver. Concerning the genotype effect, there was no evidence of substantial genotypic difference in lipid metabolism, LC-PUFA synthesis and flesh fatty acid profile when fed with vegetable oils. The minor reduction in plasma glucose and triglyceride levels in the F line was linked to potentially higher glucose and lipid uptake in the muscle. Overall, these data emphasize the importance of dietary macro-nutrient interface in evolving fish nutrition strategies.

## Introduction

Long chain polyunsaturated fatty acids (LC-PUFA) are indispensable in human nutrition due to their vital role in health, development and functionality of several organs. Compared to other food products, fish is the most salubrious source of n-3 LC-PUFA besides providing high quality protein and essential micronutrients [1], [2]. Nevertheless, the fatty acid content of the fish varies depending on the species, genotype, dietary and environmental factors [3]–[5].

At present, an increasing proportion of fish for human consumption comes from aquaculture, where formulation of feeds with fish meal and fish oil ensures n-3 LC-PUFA rich meat production. Parallel to the expansion of aquaculture the demand for these marine ingredients also increases, but global supply has stagnated and the reduction fishery that provides it is perceived as unsustainable and over-exploitative of natural resources [6]. When addressing the imminent shortage, replacement of fish oil by vegetable oils challenges the maintenance of the recognized health benefits due to n-3 LC-PUFA content. Several studies have shown that complete or partial replacement of fish oil with single vegetable oil such as rapeseed oil, linseed oil, palm oil or with a vegetable oil blend has limited effect on growth but marked consequences on lipid metabolism, tissue lipid composition and associated factors such as digestibility, fatty acid catabolism, lipid transport and uptake, lipogenesis, fatty acid desaturation and elongation, and eicosanoid synthesis [4], [7], [8]. Mostly, dietary fatty acid composition is reflected in the flesh and lipid stores of the fish. However, the magnitude of the change is dependent on the species, fatness and tissues [4].

Salmonid fishes have the capacity to convert dietary essential C_18_ PUFA to physiologically essential C_20_ and C_22_ PUFA through a series of alternating desaturation and chain elongation reactions mediated by microsomal desaturase and elongase enzymes, together with the peroxisomal Sprecher shunt [9]. Molecular studies have elucidated the genetics of these processes through the cloning and characterisation of the genes, thus enabling the study of their expression, regulation and manipulation [10]-[15]. Vegetable oils rich in 18:3n-3 are known to enhance fatty acid elongation and desaturation processes, both at molecular and enzymatic level [15], [16]. Also, one instance of an increase in *Δ*6 desaturase expression linked to dietary carbohydrate intake has been reported in rainbow trout [10]. Though exact mechanisms are unknown, the well known association of carbohydrate intake and increased fatty acid synthesis through the supply of reducing equivalents and substrates [17] may extend also to fatty acid bioconversion. Thus we hypothesized that in a low n-3 LC-PUFA (vegetable oil) dietary environment; carbohydrate intake may enhance the stimulation of fatty acid bioconversion pathway leading to beneficial flesh lipid composition.

We tested this hypothesis in two genotypes of rainbow trout because genetic makeup was found to influence the lipid deposition and metabolism in fish through a highly heritable genetic component governing the capacity to synthesize and/or deposit LC-PUFA, particularly when fed diets low in these fatty acids [5], [18], [19]. The two experimental rainbow trout lines, namely fat line (F) and lean line (L), were developed through divergent selection for muscle fat content using a non-invasive technique [20]. Previous studies revealed that the two lines consistently differed in growth and hepatic intermediary metabolism, albeit under different dietary regimes [21]–[23]. Compared to the L line, the F line was found to have lower plasma glucose levels 24 h after the last meal [22]; reduced hepatic fatty acid oxidation and enhanced glycolysis in liver and muscle [21]; higher lipogenic potential coupled with higher liver glycogen content with an increase in dietary carbohydrate intake [23], all of which suggest a better ability of the F line to metabolise and store glucose. Importantly, the F line also exhibited a prospective genetic pre-disposition for higher LC-PUFA synthesis, both in the liver and intestine [23], [24]. These features make the two lines unique models to study genotypic response to dietary manipulations focusing on sustainable feeds.

The aim of the present study was therefore to investigate the impact of dietary carbohydrate on the metabolism and tissue lipid composition of these two trout lines, when fed vegetable oil based diets containing low levels of n-3 LC-PUFA. At the phenotypic level, we measured postprandial plasma metabolite levels and the fatty acid composition of the muscle and liver. At the molecular level, we analysed the gene expression of intestinal markers of glucose and lipid absorption; markers of carbohydrate (glycolysis and gluconeogenesis) and lipid (lipogenesis and β-oxidation) metabolism in the liver, muscle and adipose tissue; transcriptional factors of fatty acid metabolism; key enzymes of the fatty acid bioconversion pathway in the liver and intestine; and markers of glucose and fatty acid uptake in the muscle.

## Materials and Methods

### Ethics statement

The experiment was carried out in strict accordance with EU legal frameworks relating to the protection of animals used for scientific purposes (Directive 2010/63/EU) and guidelines of the French legislation governing the ethical treatment of animals (Decree no. 2001-464, May 29th, 2001). It was approved by the ethics committee of INRA (INRA 2002-36, April 14, 2002). The INRA experimental station is certified for animal services under the permit number A64.495.1 by the French veterinary services, which is the competent authority.

### Experimental fish and diets

The study was conducted with juvenile rainbow trout (*Oncorhynchus mykiss*, Walbaum) from two experimental lines, namely fat line (F) and lean line (L), obtained after four generations of divergent selection for high or low muscle fat content using a non-invasive method (Distell Fish Fatmeter, Fauldhouse, West Lothian, UK), as detailed by Quillet et al. [20]. Fish were reared in the INRA flow-through experimental facilities at Donzacq (Landes, France) at a constant water temperature of 17°C, under natural photoperiod during the months of April-July. They were fed a standard commercial trout diet during the acclimatisation period (T-3P classic, Skretting, France). Two experimental diets, namely VOC- (vegetable oil based diet without carbohydrate) and VOC+ (vegetable oil based diet with carbohydrate) were prepared in our own facilities (INRA, Donzacq, Landes, France) as extruded pellets. The vegetable oil mix used in the diet formulation comprised of linseed oil, palm oil and rapeseed oil in the ratio 50∶30∶20, respectively. Gelatinized starch was included as the carbohydrate source. The VOC- diet contained 1.8% starch, whereas the VOC+ diet contained 17.1% starch (Table 1). The increase in dietary carbohydrate content was accompanied by a decrease in the proportion of protein in the VOC+ diet.

**Table 1:** Composition of diets.

	VOC-	VOC+
*Ingredients*, %		
Fish meal^1^	60	60
Wheat gluten^2^	20	0
Gelatinized starch^2^	0	20
Vegetable oil mix^3^	18	18
Mineral mix^4^	1	1
Vitamin mix^5^	1	1
*Analytical composition*		
Dry matter, %	94.0	94.4
Protein, % DM	58.6	43.0
Lipid, % DM	25.4	25.7
Starch, % DM	1.8	17.1
Energy, kJ/g DM	24.9	24.4
Ash, % DM	9.3	8.6

VOC-, diet without carbohydrate; VOC+, diet with carbohydrate; DM, dry matter.

1 Sopropeche, Boulogne-sur-Mer, France

2 Roquette, Lestrem, France

3 Linseed/Palm/Rapeseed oil in the ratio 50∶30∶20 (Daudruy, Dunkerque, France)

4 Supplied the following (kg^−1^ diet): calcium carbonate (40% Ca) 2.15 g, magnesium oxide (60% Mg) 1.24 g, ferric citrate 0.2 g, potassium iodide (75% I) 0.4 mg, zinc sulphate (36% Zn) 0.4 g, copper sulphate (25% Cu) 0.3 g, manganese sulphate (33% Mn) 0.3 g, dibasic calcium phosphate (20% Ca, 18% P) 5 g, cobalt sulphate 2 mg, sodium selenite (30% Se) 3 mg, potassium chloride 0.9 g, Sodium chloride 0.4 g.

5 Supplied the following (kg^−1^ diet): DL-a tocopherol acetate 60 IU, sodium menadione bisulphate 5 mg, retinyl acetate 15000 IU, DLcholecalciferol 3000 IU, thiamin 15 mg, riboflavin 30 mg, pyridoxine 15 mg, vit. B_12_ 0.05 mg, nicotinic acid 175 mg, folic acid 500 mg, inositol 1000 mg, biotin 2.5 mg, calcium panthotenate 50 mg, choline chloride 2000 mg.

### Feeding trial and sampling procedure

Fish of each line were distributed into six tanks of 30 animals (mean weight 120±1.5 g). Triplicate groups of each genotype were fed either the VOC- or the VOC+ diet, twice a day ad-libitum for a period of 12 weeks. The fish were bulk weighed every 3 wks and counted to calculate the mean body weight. Feed intake was recorded. At the end of the trial, nine fish per group (3/tank) were randomly sampled at 2 and 8 h after the last meal. Trout were anaesthetised in diluted 2-phenoxyethanol (0.05%), individually weighed and sacrificed after collecting blood, by severing the spinal cord behind the head. Blood was removed from the caudal vein into heparinised syringes and centrifuged (3,000 *g*, 5 min), the recovered plasma was immediately frozen and kept at −20°C until analysis. Gut content of each fish was systematically checked to confirm that the fish sampled had effectively consumed the diet. Liver, dorso-ventral white muscle, intestine (midgut - region after the last pyloric caecum to the start of the distal segment of the intestine) and perivisceral adipose tissue were dissected, weighed, immediately frozen in liquid nitrogen and kept at −80°C until analysis. The weight of liver and viscera was used to calculate the hepato/viscero-somatic index [in (%) = 100× (X weight/body weight), where X is liver or viscera]. Each tank with 30 fish represented one experimental unit to calculate feed intake and body weight. Daily feed intake (in dry weight basis) was calculated as the total amount of feed supplied and ingested (kg) divided by the mean biomass over the trial [(initial biomass + final biomass)/2, expressed in kg wet weight (WW)] and the number of days. Final body weight was calculated as the final biomass divided by the number of fish in each tank at the end of the feeding trial.

### Analytical methods

The chemical composition of the diets was analyzed using the following procedures: dry matter after drying at 105°C for 24 h, lipid content by petroleum ether extraction (Soxtherm), protein content (N×6.25) by the Kjeldahl method after acid digestion, gross energy in an adiabatic bomb calorimeter (IKA, Heitersheim Gribheimer, Germany), ash content by incinerating the samples in a muffle furnace at 600°C for 6 h and starch content by enzymatic method (InVivo labs, France). Plasma glucose (Glucose RTU, bioMérieux, Marcy l′Etoile, France), triglycerides (Triglycerides PAP 150, bioMérieux) and cholesterol (Cholesterol RTU, bioMérieux) levels were determined using commercial kits adapted to a microplate format, according to the recommendations of the manufacturer. Total plasma free amino acid levels were determined by ninhydrin reaction according to the method of Moore [25] with glycine as a standard.

### mRNA levels analysis: quantitative RT-PCR

Analyses of mRNA levels were performed on samples from the liver, white muscle, intestine and perivisceral adipose tissue of fish sampled 8 h after the last meal. This time point corresponds to the post-prandial peak of nutrient absorption in juvenile rainbow trout reared at 17°C. Tissue samples from six individual fish per experimental condition (chosen based on homogeneity of plasma glucose levels) were used as biological replicates. Total RNA was extracted using TRIzol (Invitrogen, Carlsbad, CA) according to the manufacturer's recommendations, quantified by spectrophotometry (absorbance at 260 nm) and integrity was controlled by agarose gel electrophoresis. One microgram of the resulting total RNA was reverse transcribed into cDNA using the SuperScript III RNaseH- reverse transcriptase kit (Invitrogen) and random primers (Promega, Charbonniéres, France), according to the instructions of each manufacturer. Quantification of target gene expression levels were carried out in an iCycler iQ real-time PCR detection system (Bio-Rad) using iQ SYBR green supermix and specific primers (Table 2). PCR was performed using 5 µl of the diluted cDNA (1∶50) mixed with 200 nM of each primer in a final volume of 15 µl. The PCR protocol was initiated at 95°C for 90 s for initial denaturation of the cDNA and activation of the hot-start iTaq TM DNA polymerase, followed by a two-step amplification program (20 s at 95°C and 30 s at specific primer hybridization temperature) repeated 40 times. At the end of the last amplification cycle, melting curves (temperature gradient at 0.5°C/10 s from 55 to 94°C) were systematically monitored to confirm the specificity of the amplification reaction. Each PCR run included replicate samples (duplicate of reverse transcription and PCR amplification, respectively) and negative controls (reverse transcriptase and RNA free samples, respectively). The qPCR assay was optimized with a linear standard curve (R^2^>0.985) and checked for consistency across replicates. PCR reaction efficiency for each run was estimated based on the slope of the 5 points standard curve obtained with serial dilution of pooled sample cDNAs. E values ranging from 1.85 to 2.05 were considered to be acceptable. The transcripts analyzed were sodium dependent glucose co-transporter type 1 (SGLT1), glucose facilitative transporter type 2 and 4 (GLUT2, GLUT4) for glucose transport/uptake; microsomal triglyceride transfer protein (MTP), apolipoproteins B, A1 and A4 (ApoB, ApoA1, ApoA4) for chylomicron synthesis; lipoprotein lipase (LPL; EC 3.1.1.34), very low density lipoprotein receptor (VLDLR) and fatty acid translocase (CD36) for lipid uptake; glucokinase (GK; EC 2.7.1.2) and hexokinase (HK; EC 2.7.1.1) for glycolysis; glucose 6-phosphatase (G6Pase; EC 3.1.3.9) for gluconeogenesis; glucose 6-phosphate dehydrogenase (G6PD; EC 1.1.1.49), adenosine triphosphate citrate lyase (ACLY; EC 2.3.3.8), acetyl coA carboxylase (ACC; EC 6.4.1.2), fatty acid synthase (FAS; EC 2.3.1.85) and Δ9 fatty acyl desaturase (D9D; EC 1.14.19.1) for lipogenesis; Δ6 fatty acyl desaturase (D6D; EC 1.14.19.3), elongation of very long chain fatty acids like 5 and 2 (Elovl5, Elovl2; EC 2.3.1.199) for fatty acid bioconversion; carnitine palmitoyl transferase (CPT1; EC 2.3.1.21) for fatty acid oxidation; sterol regulatory element binding protein 1c (SREBP1c) and peroxisome proliferator-activated receptor (PPAR) for transcriptional regulatory factors. When different isoforms of a gene were known in rainbow trout (as for G6Pase, CPT1 and PPAR), gene expression analysis was performed on each isoform. 18 S ribosomal RNA (18 S) was employed as a non-regulated reference gene and it was found to be stably expressed in this study. Relative quantification of target gene expression was performed using the mathematical model described by Pfaffl [26], after correcting for reaction efficiency (efficiency-calibrated model).

**Table 2:** Primer sequences.

Gene	Foward primer (5′ - 3′)	Reverse primer (5′ - 3′)	Database and Accession No.	Annealing Temperature, ^o^C	Amplicon size, bp
18 S	CGGAGGTTCGAAGACGATCA	TCGCTAGTTGGCATCGTTTAT	GenBank AF308735	56	62
SGLT1	TCTGGGGCTGAACATCTACC	GAAGGCATAACCCATGAGGA	GenBank AY210436	59	154
GLUT2	GTGGAGAAGGAGGCGCAAGT	GCCACCGACACCATGGTAAA	GenBank AF321816	59	227
MTP	CTCACTGACCACTCCCAGGT	ATGGCTCCCTTGTTGTTGAC	GenBank BX860503	55	152
ApoB	CCCTGTCTTCAAAGCCACAC	GTGGCGGGAGACACTCATAG	GenBank CA383905	55	196
ApoA1	CGCAGGTACCCAGGCTTTTC	AATGGACCTCTGTGCGGTCA	GenBank AF042218	59	115
ApoA4	AGCTGGGACAGGATGTCAAT	AGACGCTCTCTCAGCACCTC	GenBank CA363690	55	148
GK	TGAAGGATCAGAGGTGGGTGATT	GAAGGTGAAACCCAGAGGAAGC	GenBank AF135403	59	253
HK1	CTGGGACGCTGAAGACCAGA	CGGTGCTGCATACCTCCTTG	GenBank AY864082	59	159
G6Pase1	CTCAGTGGCGACAGAAAGG	TACACAGCAGCATCCAGAGC	Sigenae tcay0019b.d.18_3.1.s.om.8	55	77
G6Pase2	TAGCCATCATGCTGACCAAG	CAGAAGAACGCCCACAGAGT	GenBank AF120150	55	82
G6PD	CTCATGGTCCTCAGGTTTG	AGAGAGCATCTGGAGCAAGT	GenBank CA351434	59	176
ACLY	CTGAAGCCCAGACAAGGAAG	CAGATTGGAGGCCAAGATGT	GenBank CA349411.1	60	149
ACC	TGGAGCTCTACGCAGACAGA	CTCCGGTGTACCAAGCTGTT	Sigenae tcbk0010c.b.21_5.1.om.4	55	152
FAS	TGATCTGAAGGCCCGTGTCA	GGGTGACGTTGCCGTGGTAT	Sigenae tcab0001c.e.06_5.1.s.om.8	60	161
D9D	GCCGTCCGAGGGTTCTTCTT	CTCTCCCCACAGGCACCAAG	GenBank FP323026	60	204
SREBP1c	GACAAGGTGGTCCAGTTGCT	CACACGTTAGTCCGCATCAC	GenBank CA048941.1	60	59
D6D	AGGGTGCCTCTGCTAACTGG	TGGTGTTGGTGATGGTAGGG	Genbank AF301910	59	175
Elovl2	TGTGGTTTCCCCGTTGGATGCC	ACAGAGTGGCCATTTGGGCG	Sigenae FYV3OTN01A4WMI.s.om.10	59	146
Elovl5	GAACAGCTTCATCCATGTCC	TGACTGCACATATCGTCTGG	Genbank AY605100	59	149
CPT1a	TCGATTTTCAAGGGTCTTCG	CACAACGATCAGCAAACTGG	GenBank AF327058	55	166
CPT1b	CCCTAAGCAAAAAGGGTCTTCA	CATGATGTCACTCCCGACAG	GenBank AJ606076	55	149
CPT1c	CGCTTCAAGAATGGGGTGAT	CAACCACCTGCTGTTTCTCA	GenBank AJ619768	59	187
CPT1d	CCGTTCCTAACAGAGGTGCT	ACACTCCGTAGCCATCGTCT	GenBank AJ620356	59	154
PPARα*	CTGGAGCTGGATGACAGTGA	GGCAAGTTTTTGCAGCAGAT	GenBank AY494835	54	195
PPARβ*	CTGGAGCTGGATGACAGTGA	GTCAGCCATCTTGTTGAGCA	GenBank AY356399	60	195
PPARγ*	GACGGCGGGTCAGTACTTTA	ATGCTCTTGGCGAACTCTGT	Genbank CA345564	60	171
GLUT4	GGCGATCGTCACAGGGATTC	AGCCTCCCAAGCCGCTCTT	GenBank AF247395	60	207
LPL	TAATTGGCTGCAGAAAACAC	CGTCAGCAAACTCAAAGGT	GenBank AJ224693	59	164
VLDLR	GTTTTGGACAGATGGGAGA	AGCCTTCTCATTGCACCAGT	GenBank BX077158	60	160
CD36	CCACTGAAGTTGAGCCATGA	TGCTAGACTCATGCCGTGTC	GenBank BX300637	60	121

* Sánchez-Gurmaches et al. [69]

### Total lipids and fatty acids analyses

Total lipids of the muscle and liver samples were extracted according to Folch et al. [27], using dichloromethane instead of chloroform as the solvent and quantified gravimetrically. Fatty acid composition was determined on the total lipid extract. Fatty acid methyl esters were prepared by acid-catalysed transmethylation of total lipids using boron trifluoride (BF3) in methanol (14%) according to Shantha and Ackman [28]. They were analysed in a Varian 3800 gas chromatograph (Varian, Les Ulis, France). The chromatograph was equipped with a DB Wax fused silica capillary column (30 m×0.25 mm internal diameter, film thickness 0.25 µm; J & W Scientific, Folsom, CA, USA). Injection was made in a split mode (ratio 1∶40) with 1 µL injected. Injector and flame ionization detector temperatures were 260 and 250°C, respectively. Helium was used as carrier gas (1 ml/min) and the thermal gradient during separation was 100 to 180°C at 8°C/min, 180 to 220°C at 4°C/min and a constant temperature of 220°C during 20 min. Fatty acid methyl esters were identified by comparison with known standard mixtures (Sigma189-19, St Louis, MO, USA) and quantified using the STAR computer package (Varian).

### Statistical analysis

The results are presented as means ± s.d. The effect of diet, line, and diet x line interaction on the different parameters was tested using statistical software (StatView 5.0, SAS Institute, Cary, NC) by means of a two-way analysis of variance (ANOVA) with diet and line as independent variables. Post-hoc comparisons were made using a Student–Newman–Keuls multiple range test and differences were considered statistically significant at *P*<0.05. When diet x line interaction was significant, means were compared using one way ANOVA.

## Results

Feed intake was slightly higher in the L line than the F line, irrespective of the diet (Table 3). Correspondingly, the L line fish were heavier at the end of the feeding trial, than those of the F line. The VOC+ diet was associated with an increase in the weight of liver and viscera in both lines. Hepato-somatic index was higher in the F line, regardless of the diet.

**Table 3:** Feed intake, morphological indices and tissue lipid content.

	VOC-		VOC+		*P values*		
	Fat	Lean	Fat	Lean	Diet	Line	D*L
Feed intake, g/kg/day	12.9±0.2	13.2±0.2	12.5±0.4	13.2±0.3	0.32	0.02	0.29
Final body weight, g	216.7±28.4	268.9±6.0	210.1±12.6	269.4±3.1	0.74	3×10^−4^	0.71
Hepato-somatic index, %	1.5±0.3	1.4±0.2	1.7±0.2	1.6±0.3	2×10^−4^	0.03	0.58
Viscero-somatic index, %	11.5±2.0	11.8±3.0	12.3±1.8	13.4±1.1	0.006	0.15	0.35
Muscle lipid, %	7.9±1.2	4.2±0.8	8.2±1.2	4.7±0.8	0.37	<10^−4^	0.83
Liver lipid, %	5.3±0.3^a^	5.2±0.3^a^	5.1±0.5^a^	4.5±0.2^b^	2×10^−4^	0.001	0.02

The data are represented as means ± s.d. (*N* = 3 tanks for feed intake and body weight estimation; *N* = 9 individuals for morphological indices estimation; *N* = 6 individuals for tissue lipid analysis) and were analysed by two-way ANOVA (*P*<0.05) followed by Student–Newman–Keuls multiple comparison test.

### Plasma metabolite levels

Plasma metabolite levels at 2 and 8 h after the meal are summarized in table 4. The intake of VOC+ diet caused a moderate but significant hyperglycemia in both lines, as compared to the VOC- diet. Temporal differences were not observed in any group. Nevertheless, the F line fish seemed to have a slightly better control of glycemia at 8 h after the carbohydrate rich meal. Triglyceride levels were found to be elevated in the plasma at 8 h in all the groups, particularly more in the L line than the F line, regardless of the diet. Dietary carbohydrate intake (VOC+) was linked to an increase in plasma cholesterol levels at 2 h after the meal.

**Table 4:** Postprandial plasma metabolites.

Plasma metabolites		VOC-		VOC+		*P values*		
		Fat	Lean	Fat	Lean	Diet	Line	D*L
Glucose (mmol/L)	2 h	4.4±0.7	4.7±0.6	5.9±0.9	5.7±1.1	<10^−4^	0.95	0.41
	8 h	5.1±0.8	5.3±0.8	5.7±1.0	7.5±2.4	0.006	0.05	0.09
Triglycerides (mmol/L)	2 h	1.2±0.7 ^a^	1.0±0.5 ^a^	1.6±0.9	1.5±0.9 ^a^	0.12	0.54	0.82
	8 h	2.2±1.2 ^b^	3.2±1.7 ^b^	2.0±1.3	3.6±1.5 ^b^	0.83	0.01	0.61
Cholesterol (mmol/L)	2 h	4.2±1.1	4.5±0.5	5.0±1.1	5.7±1.3	0.005	0.13	0.47
	8 h	4.4±1.2	4.9±1.6	5.0±1.5	5.2±1.3	0.33	0.43	0.81
Free amino acids (mg Eq. Glycine/ml)	2 h	0.5±0.1	0.5±0.06	0.5±0.08 ^b^	0.44±0.06 ^b^	0.08	0.24	0.74
	8 h	0.5±0.1	0.5±0.07	0.4±0.04 ^a^	0.36±0.08 ^a^	0.003	0.19	0.70

Data are presented as means ± s.d. (*N* = 9 individuals). At each postprandial time, the effect of diet, line and interaction were analysed by two-way ANOVA (*P*<0.05) followed by Student–Newman–Keuls multiple comparison test. Within each dietary treatment (column wise), significant differences in postprandial kinetics (2 h, 8 h after the last meal) are represented with different superscripts ^a,b^ (one-way ANOVA, *P*<0.05).

### mRNA levels of target genes

**Figure 1:**
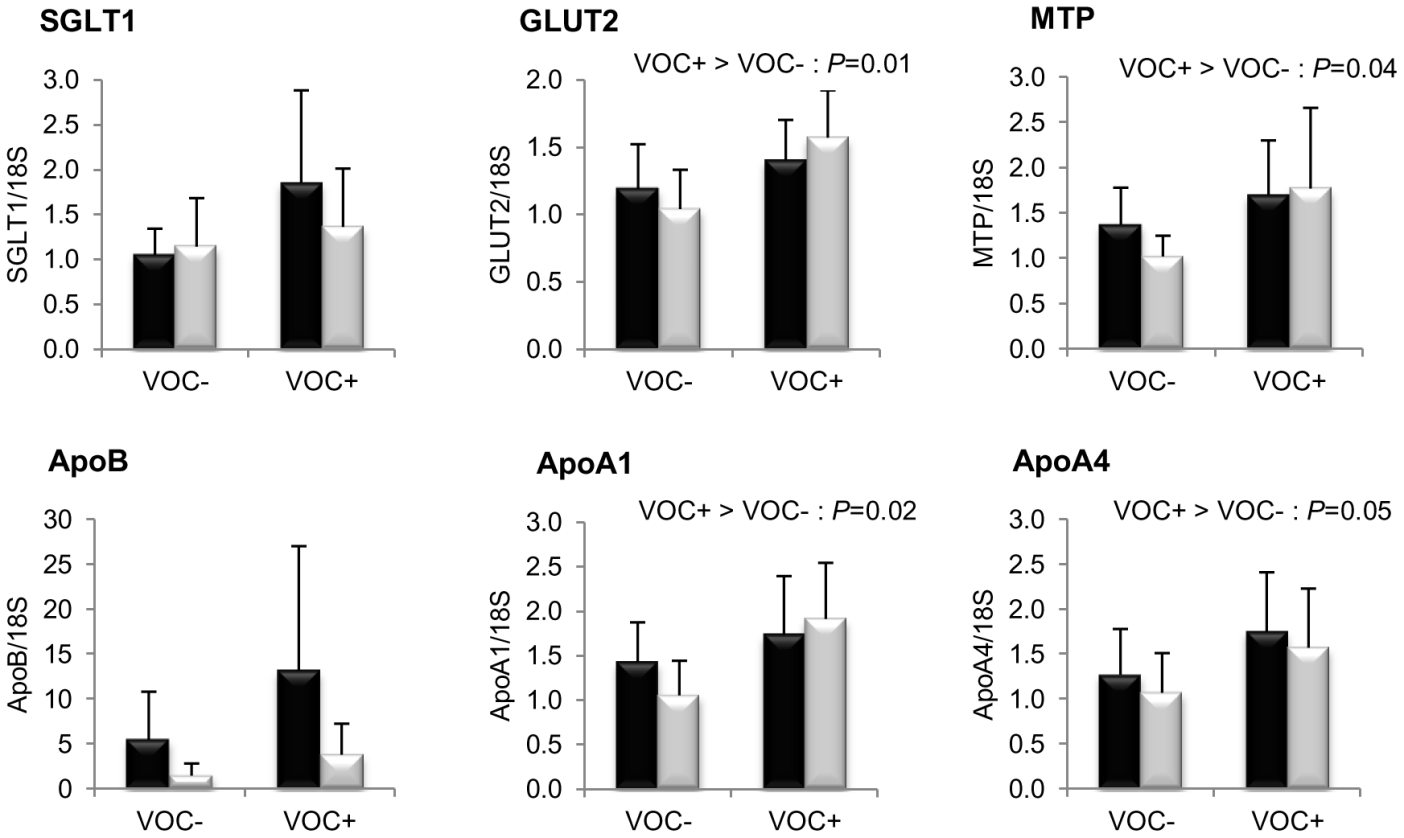
Gene expression of intestinal membrane glucose transporters and proteins involved in chylomicron assembly. mRNA levels of sodium-dependent glucose co-transporter type 1 (SGLT1), glucose facilitative transporter type 2 (GLUT2), microsomal triglyceride transfer protein (MTP), apolipoprotein B (ApoB), apolipoprotein A1 (ApoA1) and apolipoprotein A4 (ApoA4) were measured using real-time quantitative RT-PCR in the intestine of rainbow trout from a fat line (F; black bar) and a lean line (L; grey bar) fed a diet without (VOC-) or with (VOC+) carbohydrate, 8 h after the last meal. Expression values are normalized by 18 S ribosomal RNA (18 S) expressed transcripts. Relative fold difference between treatments are presented as means + s.d. (*N* = 6 individuals) and were analyzed using two-way ANOVA followed by Student–Newman–Keuls test for multiple comparison. Differences were considered significant at *P*<0.05

We analyzed the expression of several marker genes encoding the proteins involved in absorption, metabolism and uptake of glucose and fatty acids, to elucidate the effect of dietary carbohydrate on the molecular regulation of intermediary metabolism in the two trout lines fed a vegetable oil based diet lacking n-3 LC-PUFA. Representing intestinal nutrient absorption, the results of glucose transporters and chylomicron assembly associated proteins are shown in figure 1. Among the two glucose transporters studied, GLUT2 transcripts were significantly enhanced by dietary starch intake, but such an induction was not statistically significant for SGLT1 (*P* = 0.09). The mRNA levels of key proteins involved in intestinal chylomicron synthesis such as MTP, ApoA1 and ApoA4 were higher in the VOC+ dietary group. There was no difference between the two lines for these markers of nutrient absorption.

**Figure 2:**
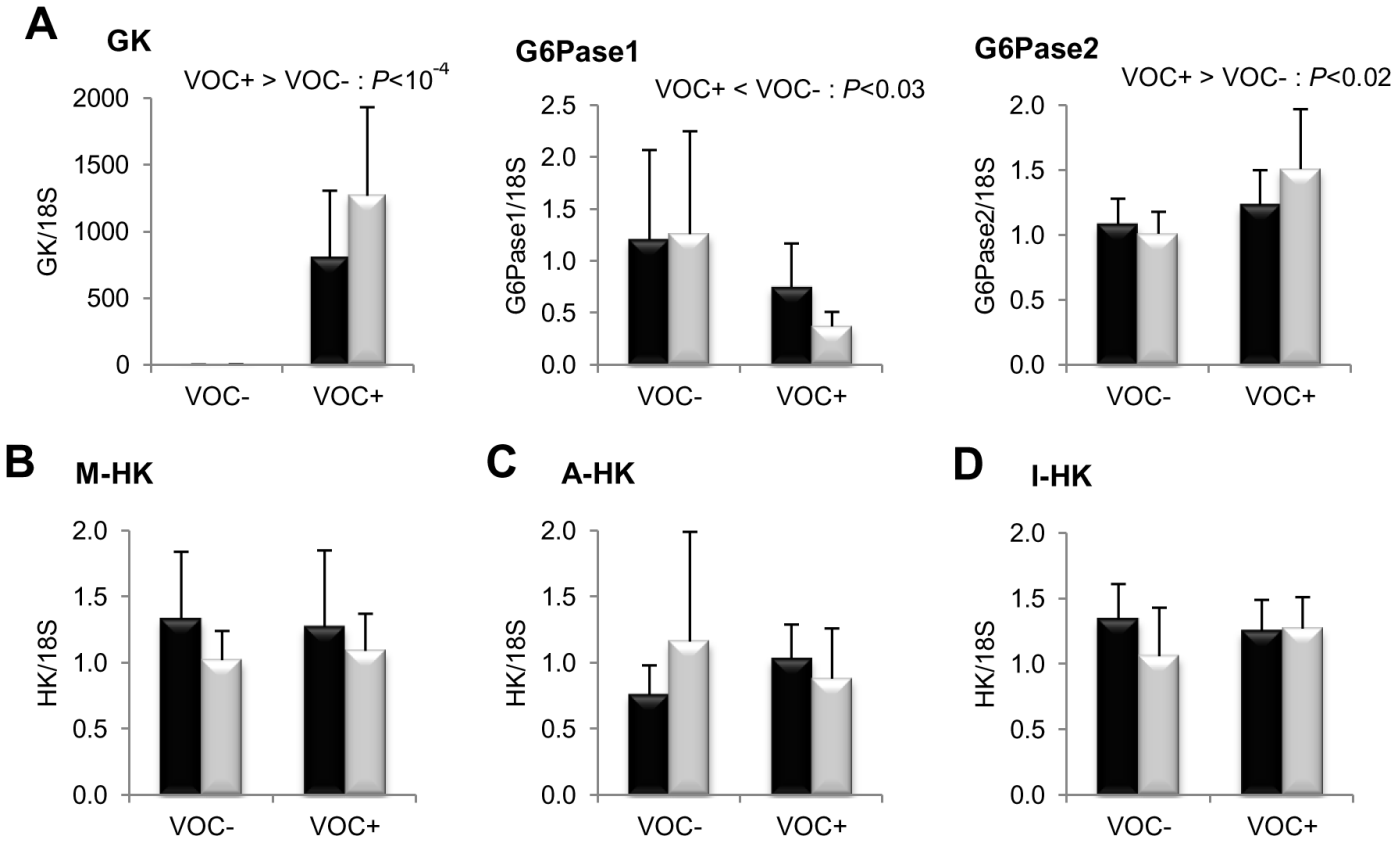
Gene expression of selected glycolytic and gluconeogenic enzymes. mRNA levels of glucokinase (GK), glucose-6-phosphatase isoform 1 (G6Pase1), isoform 2 (G6Pase2) and hexokinase (HK) were measured using real-time quantitative RT-PCR in the liver (row A); muscle (B), adipose tissue (C) and intestine (D) of rainbow trout from a fat line (F; black bar) and a lean line (L; grey bar) fed a diet without (VOC−) or with (VOC+) carbohydrate, 8 h after the last meal. Expression values are normalized by 18 S ribosomal RNA (18 S) expressed transcripts. Relative fold difference between treatments are presented as means + s.d. (*N* = 6 individuals) and were analyzed using two-way ANOVA followed by Student–Newman–Keuls test for multiple comparison. Differences were considered significant at *P*<0.05

**Figure 3:**
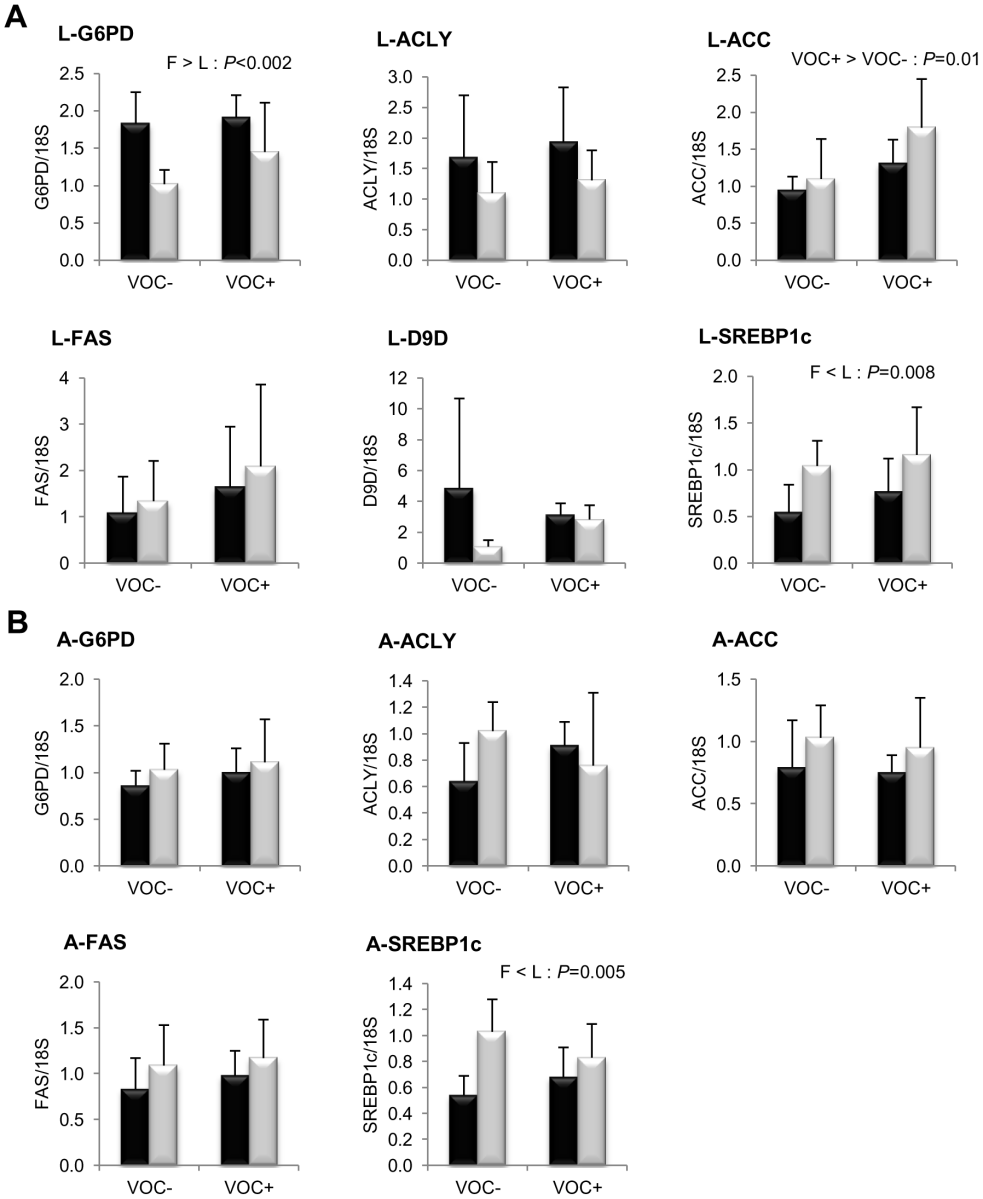
Gene expression of selected enzymes and transcription factor involved in NADPH generation and lipogenesis. mRNA levels of glucose 6-phosphate dehydrogenase (G6PD), ATP citrate lyase (ACLY), acetyl coA carboxylase (ACC), fatty acid synthase (FAS), 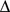
9 fatty acyl desaturase (D9D) and sterol regulatory element binding protein 1-like (SREBP-1c) were measured using real-time quantitative RT-PCR in the liver (A - two rows) and adipose tissue (B - two rows) of rainbow trout from a fat line (F; black bar) and a lean line (L; grey bar) fed a diet without (VOC−) or with (VOC+) carbohydrate, 8 h after the last meal. Expression values are normalized by 18 S ribosomal RNA (18 S) expressed transcripts. Relative fold difference between treatments are presented as means + s.d. (*N* = 6 individuals) and were analyzed using two-way ANOVA followed by Student–Newman–Keuls test for multiple comparison. Differences were considered significant at *P*<0.05

The impact of dietary starch intake on glucose metabolism is illustrated in figure 2. The glycolytic enzyme GK exhibited a strong transcriptional induction of several hundred fold in the liver of fish from both lines, when fed the VOC+ diet. However, no such response to dietary starch intake was noticed in the HK transcripts of muscle, adipose tissue and intestine. Isoforms of the gluconeogenic enzyme G6Pase showed differential dietary regulation in the liver, VOC+ diet down-regulated G6Pase1 mRNA levels, but up-regulated G6Pase2. The only genotypic variation observed in glucose metabolism, was in the liver transcript levels of the pentose pathway enzyme G6PD, which was higher in the F line than the L line (Fig. 3).

**Figure 4:**
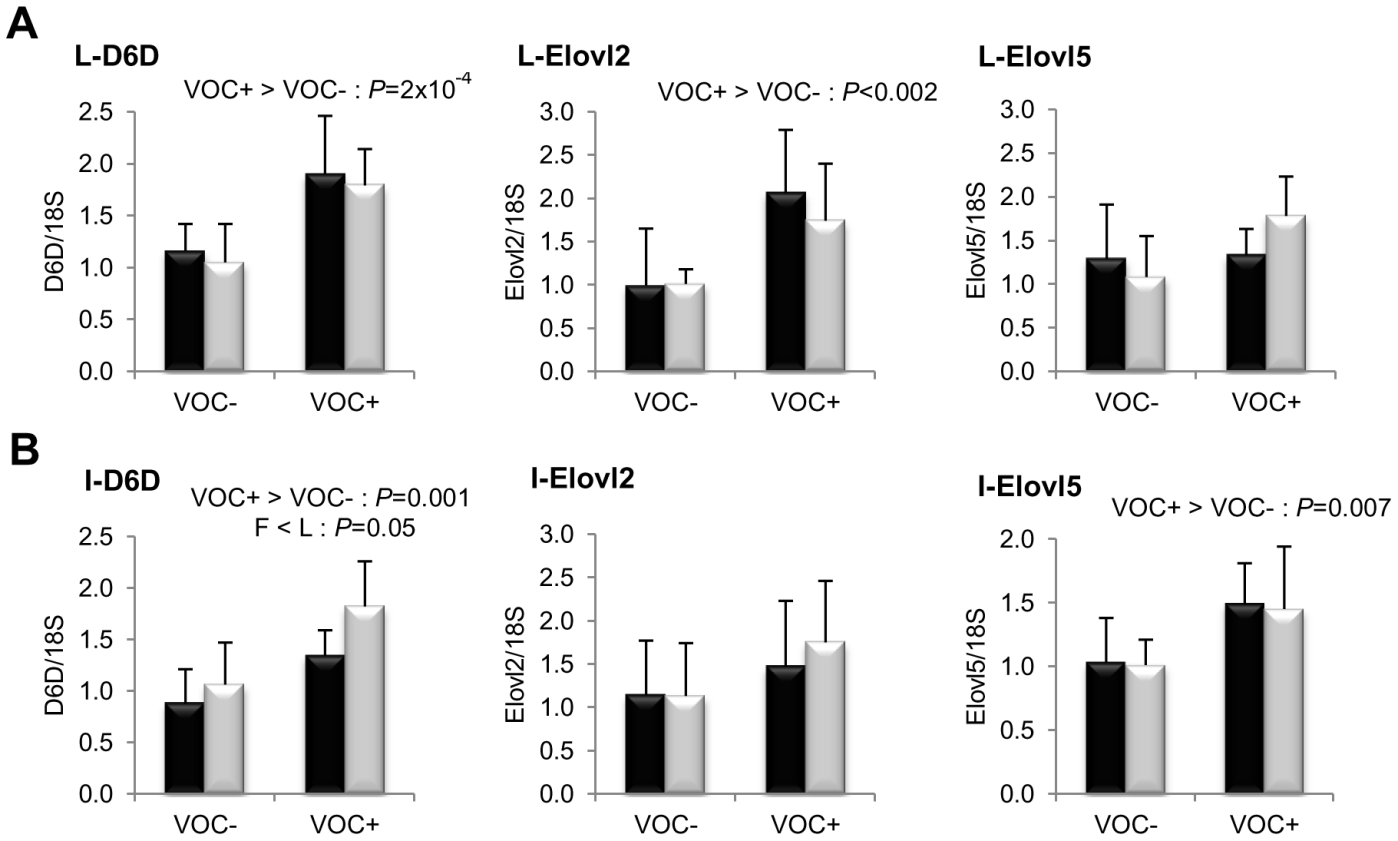
Gene expression of selected enzymes involved in fatty acid bioconversion. mRNA levels of 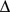
6 fatty acyl desaturase (D6D), elongation of very long chain fatty acids like-2 (Elovl2) and elongation of very long chain fatty acids like-5 (Elovl5) were measured using real-time quantitative RT-PCR in the liver (row A) and intestine (row B) of rainbow trout from a fat line (F; black bar) and a lean line (L; grey bar) fed a diet without (VOC−) or with (VOC+) carbohydrate, 8 h after the last meal. Expression values are normalized by 18 S ribosomal RNA (18 S) expressed transcripts. Relative fold difference between treatments are presented as means + s.d. (*N* = 6 individuals) and were analyzed using two-way ANOVA followed by Student–Newman–Keuls test for multiple comparison. Differences were considered significant at *P*<0.05

**Figure 5:**
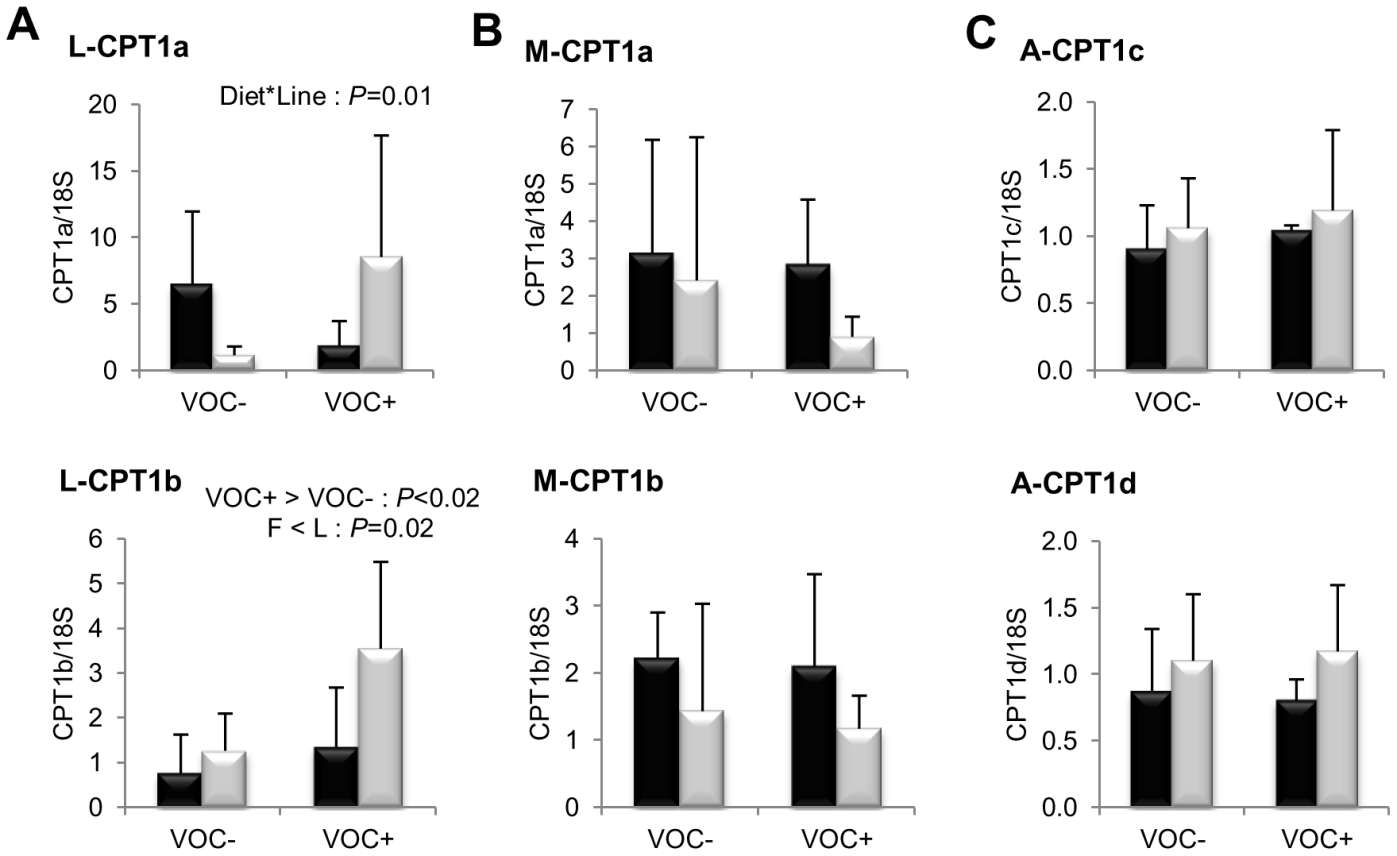
Gene expression of carnitine palmitoyl transferase (CPT1) isoforms involved in fatty acid oxidation. mRNA levels of CPT1a, CPT1b, CPT1c and CPT1d were measured using real-time quantitative RT-PCR in the liver (column A), muscle (column B) and adipose tissue (column C) of rainbow trout from a fat line (F; black bar) and a lean line (L; grey bar) fed a diet without (VOC−) or with (VOC+) carbohydrate, 8 h after the last meal. Expression values are normalized by 18 S ribosomal RNA (18 S) expressed transcripts. Relative fold difference between treatments are presented as means + s.d. (*N* = 6 individuals) and were analyzed using two-way ANOVA (*P*<0.05) followed by Student–Newman–Keuls test for multiple comparison. When interactions were significant, means were compared using one way ANOVA (*P*<0.05).

**Figure 6:**
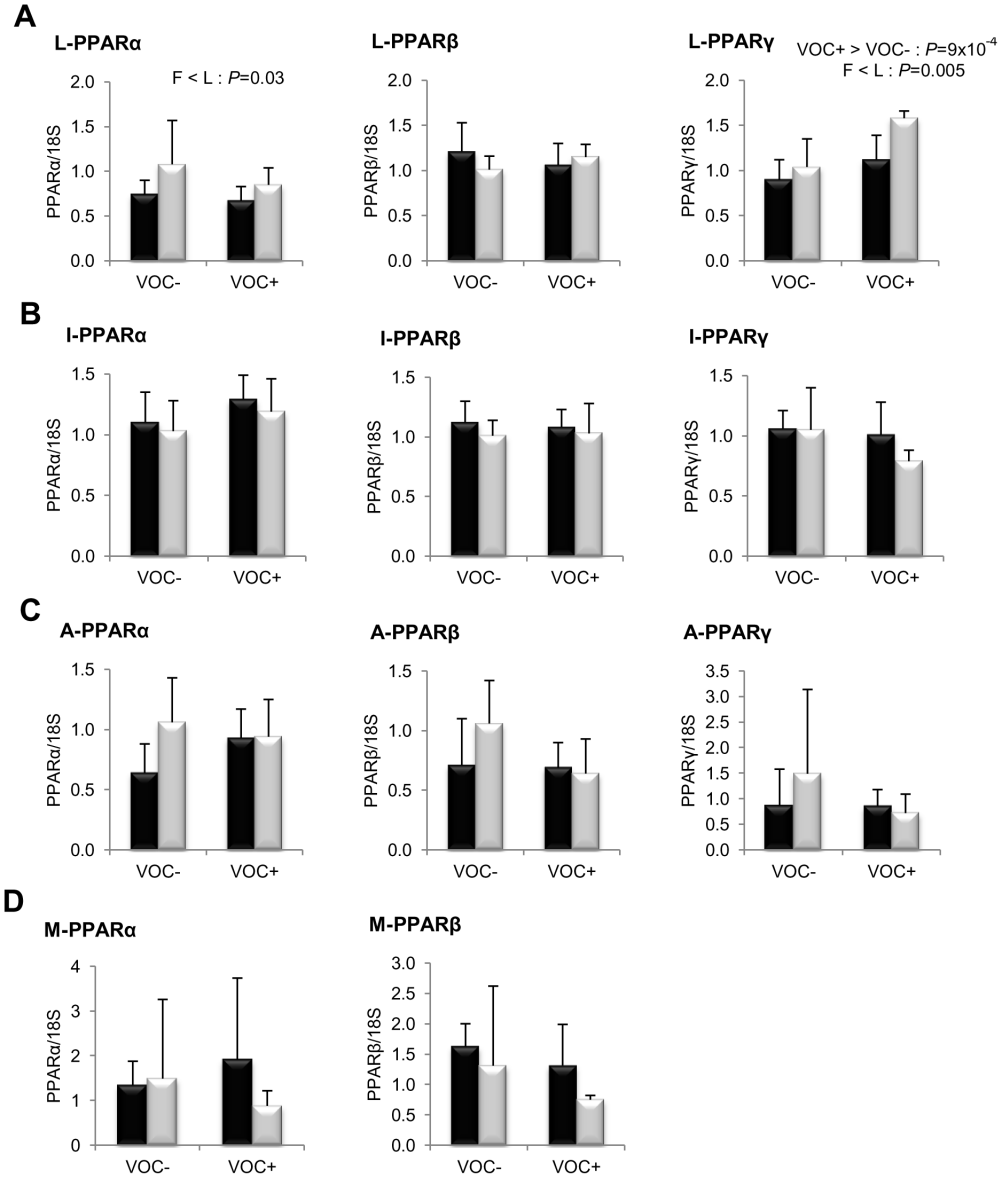
Gene expression of peroxisome proliferator activated receptor isoforms. mRNA levels of PPARα, PPARβ and PPARγ were measured using real-time quantitative RT-PCR in the liver (row A), intestine (row B), adipose tissue (row C) and muscle (row D) of rainbow trout from a fat line (F; black bar) and a lean line (L; grey bar) fed a diet without (VOC−) or with (VOC+) carbohydrate, 8 h after the last meal. Expression values are normalized by 18 S ribosomal RNA (18 S) expressed transcripts. Relative fold difference between treatments are presented as means + s.d. (*N* = 6 individuals) and were analyzed using two-way ANOVA followed by Student–Newman–Keuls test for multiple comparison. Differences were considered significant at *P*<0.05

Concerning lipogenesis (Fig. 3), the mRNA levels of the key transcription factor SREBP1c was found to be significantly elevated in the liver (*P* = 0.008) and adipose tissue (*P* = 0.005) of the L line fish, irrespective of the diets. But this genotypic difference was not reflected in the transcript levels of FAS or the other lipogenic enzymes ACLY, ACC and D9D. In both lines, the intake of VOC+ diet was found to enhance the expression of hepatic ACC, but not the other lipogenic enzymes. Regarding fatty acid bioconversion (Fig. 4), fish fed VOC+ diet displayed a distinctly higher hepatic (*P* = 0.0002) and intestinal (*P* = 0.001) expression of the D6D enzyme than those fed the VOC- diet, regardless of the genotype. Likewise, mRNA levels of hepatic Elovl2 and intestinal Elovl5 were also found to be raised in the VOC+ dietary group. The expression of D6D was higher in the intestine of the L line than the F line fish, but this was not the case for the liver D6D and for the elongases. Results pertaining to fatty acid oxidation are presented in figure 5. The two lines experienced a contrasting dietary influence on their hepatic CPT1a transcript levels leading to a disordinal interaction, where VOC+ diet up-regulates CPT1a levels in the L line and vice versa. The expression of the other hepatic isoform CPT1b was generally enhanced by the VOC+ diet, but more pronounced in the L line. The mRNA levels of the transcription factor PPAR closely corresponded to the CPT1 expression (Fig. 6). Hepatic transcripts of both PPARα and PPARγ were more abundant in the L line and the VOC+ diet was found to enhance the PPARγ levels in both lines. In the other peripheral tissues, no significant differences related to either diet or genotype were observed in the CPT1 and PPAR transcripts tested. Moreover, the expression of PPARγ was not detectable in the muscle.

**Figure 7:**
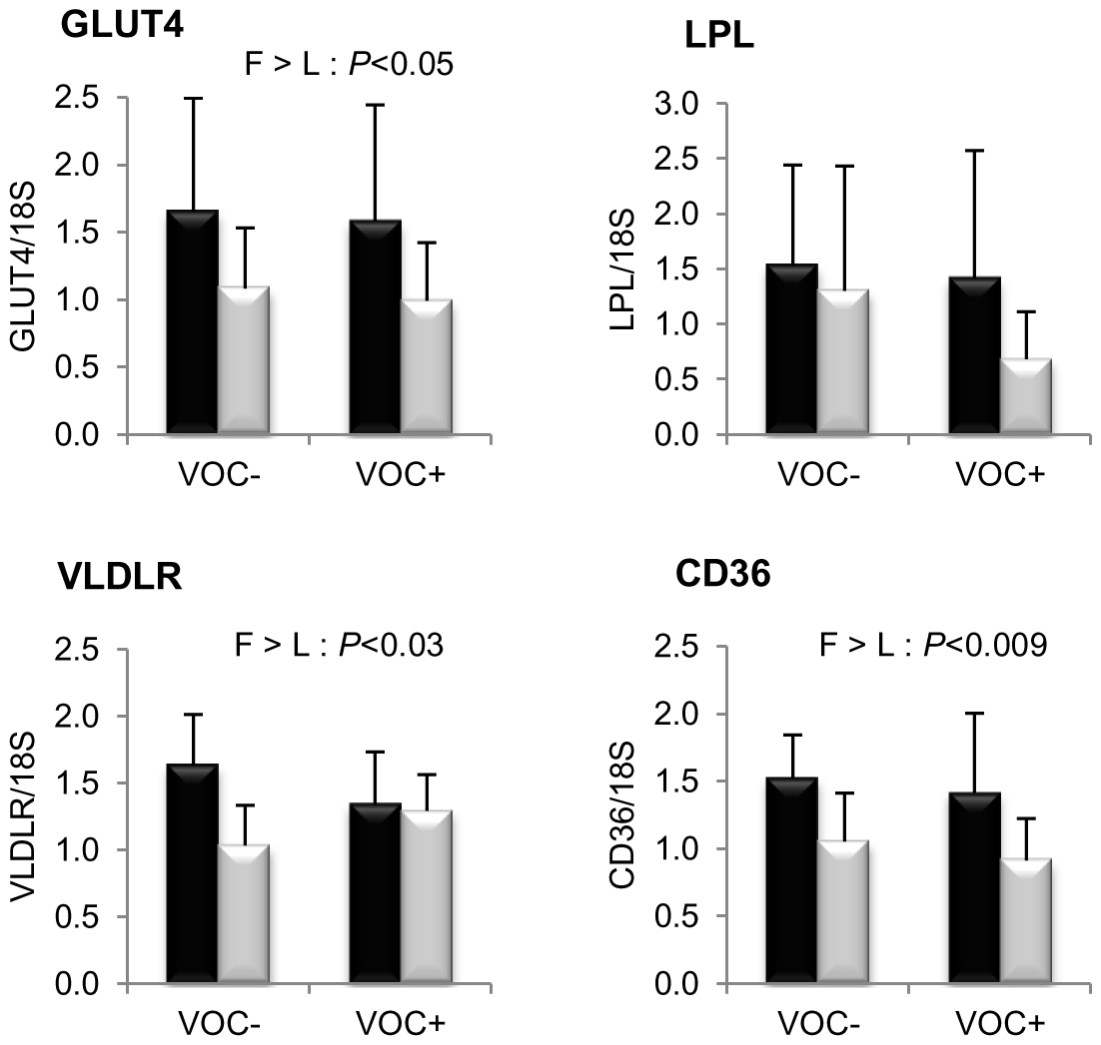
Gene expression of selected proteins involved in glucose and lipid uptake in the white muscle. mRNA levels of glucose facilitative transporter type 4 (GLUT4), lipoprotein lipase (LPL), very low density lipoprotein receptor (VLDLR) and fatty acid translocase (CD36) were measured using real-time quantitative RT-PCR in the muscle of rainbow trout from a fat line (F; black bar) and a lean line (L; grey bar) fed a diet without (VOC−) or with (VOC+) carbohydrate, 8 h after the last meal. Expression values are normalized by 18 S ribosomal RNA (18 S) expressed transcripts. Relative fold difference between treatments are presented as means + s.d. (*N* = 6 individuals) and were analyzed using two-way ANOVA followed by Student–Newman–Keuls test for multiple comparison. Differences were considered significant at *P*<0.05

Finally, mRNA levels of proteins involved in glucose and fatty acid uptake in the muscle are presented in figure 7. Transcript levels of GLUT4, VLDLR and CD36 were found to be significantly higher in the F line than the L line. However, the LPL expression was not different between the two lines. Dietary manipulation exerted no noticeable influence on the transcript abundance of these markers.

### Lipid content and fatty acid composition

Muscle lipid content was invariably higher in the F line, with no diet induced modification (Table 3). The liver lipid content witnessed an interaction between the dietary treatment and genotype, with a marked decrease in the L line fish fed the VOC+ diet. The fatty acid profiles of the two isolipidic diets are presented in table 5. The proportional content of the saturated and unsaturated fatty acids did not vary considerably between the two diets. However, there were minor differences such as slightly lower linoleic acid and higher linolenic acid in the VOC+ diet. The muscle fatty acid composition in both lines closely reflected the profile of the respective diet consumed (Table 6). There was no evidence for a positive influence of the VOC+ diet on the muscle PUFA content of the two trout lines. In terms of genotypic difference, the L line had a slightly higher proportion of PUFA (DHA) than the F line, but exactly the opposite was observed for the MUFA proportion. Considering the divergent selection criterion applied, the F line possessed a substantially larger quantity of unsaturated and saturated fatty acids per gram of muscle (Table 7). Similar to the muscle, the fatty acid profile of the liver resembled the respective dietary profile (Table 8). Furthermore, the n-3 PUFA (DHA) content of the liver exhibited a significant diet x genotype interaction, where it was typically lower in the F line fish fed the VOC- diet. Apart from this, no other differences were significant.

**Table 5:** Fatty acid profile of diets expressed as % of total fatty acids.

Fatty acids	VOC−	VOC+
SFA		
14∶0	1.4	0.8
16∶0	15.5	14.8
17∶0	0.1	0.1
18∶0	3.0	3.3
20∶0	0.3	0.3
∑SFA	20.4	19.5
MUFA		
16∶1	1.1	0.6
18∶1	28.1	31.7
20∶1	2.5	1.8
22∶1	3.2	2.2
∑MUFA	34.9	36.4
n-6 PUFA		
18∶2 n-6	13.9	13.0
20∶2 n-6	0.1	0.1
20∶4 n-6	0.1	0.1
∑n-6 PUFA	14.1	13.1
n-3 PUFA		
18∶3 n-3	23.4	25.8
18∶4 n-3	0.4	0.3
20∶3 n-3	0.1	0.05
20∶4 n-3	0.1	0.1
20∶5 n-3	1.4	0.9
22∶5 n-3	0.1	0.1
22∶6 n-3	2.9	1.9
∑n-3 PUFA	28.5	29.1
Ratios		
SFA/PUFA	0.5	0.5
n3/n6	2.0	2.2

**Table 6:** Fatty acid profile of muscle expressed as % of total fatty acids.

Fatty acids	VOC−		VOC+		*P* value		
	Fat	Lean	Fat	Lean	Diet	Line	Diet*Line
SFA							
14∶0	1.72±0.38	1.37±0.26	1.67±0.27	1.39±0.20	0.92	0.01	0.80
16∶0	12.32±1.37	12.69±1.33	11.91±0.74	12.32±0.46	0.37	0.37	0.97
17∶0	0.13±0.01	0.11±0.01	0.11±0.01	0.11±0.01	0.01	0.02	0.16
18∶0	2.97±0.30	3.23±0.13	2.90±0.22	3.11±0.22	0.33	0.02	0.77
20∶0	0.17±0.02	0.17±0.02	0.19±0.03	0.17±0.01	0.10	0.18	0.28
∑SFA	17.49±1.61	17.74±1.59	16.96±0.82	17.26±0.53	0.33	0.59	0.96
MUFA							
16∶1	2.29±0.43	1.65±0.35	2.25±0.27	1.70±0.24	0.97	3×10^−4^	0.73
18∶1	29.99±0.7 ^b^	27.50±0.9 ^a^	30.45±0.9 ^b^	29.44±1.1 ^b^	0.003	<10^−4^	0.05
20∶1	2.75±0.17	2.64±0.32	2.73±0.33	2.55±0.08	0.58	0.18	0.72
22∶1	2.31±0.36	2.40±0.39	2.56±0.43	2.32±0.21	0.56	0.64	0.27
∑MUFA	37.33±0.65	34.19±1.41	37.99±0.62	36.01±1.35	0.01	<10^−4^	0.20
n-6 PUFA							
18∶2 n-6	12.49±0.61	12.01±0.39	11.19±0.60	11.05±0.52	<10^−4^	0.17	0.45
18∶3 n-6	0.23±0.04	0.23±0.03	0.19±0.03	0.19±0.03	0.01	0.91	0.99
20∶2 n-6	0.63±0.05	0.64±0.11	0.53±0.09	0.51±0.05	0.003	0.96	0.67
20∶3 n-6	0.30±0.02 ^a^	0.36±0.06 ^b^	0.28±0.04 ^a^	0.27±0.02 ^a^	9×10^−4^	0.06	0.03
20∶4 n-6	0.35±0.03	0.40±0.02	0.33±0.04	0.37±0.04	0.04	0.002	0.72
∑n-6 PUFA	14.04±0.62	13.72±0.33	12.61±0.53	12.39±0.51	<10^−4^	0.21	0.81
n-3 PUFA							
18∶3 n-3	13.07±0.47	14.07±0.32	14.02±1.02	15.22±0.56	8×10^−4^	5×10^−4^	0.72
18∶4 n-3	1.40±0.13	1.45±0.11	1.44±0.24	1.45±0.22	0.81	0.67	0.80
20∶3 n-3	0.79±0.11	0.89±0.17	0.85±0.16	0.82±0.11	0.91	0.51	0.29
20∶4 n-3	1.00±0.16	1.15±0.27	1.13±0.18	0.92±0.08	0.49	0.75	0.03
20∶5 n-3	2.43±0.32	2.34±0.18	2.30±0.41	2.39±0.15	0.73	0.97	0.45
22∶5 n-3	1.05±0.25	0.89±0.07	1.12±0.24	0.97±0.06	0.31	0.04	0.99
22∶6 n-3	8.47±0.92	10.94±1.33	8.75±1.13	9.94±1.47	0.48	0.002	0.22
∑n-3 PUFA	28.48±1.25	32.00±1.64	29.89±0.80	31.80±1.16	0.25	<10^−4^	0.13
Ratios							
SFA/PUFA	0.41±0.05	0.39±0.04	0.39±0.02	0.39±0.01	0.72	0.33	0.63
n3/n6	2.03±0.11	2.33±0.14	2.38±0.15	2.57±0.19	10^−4^	5×10^−4^	0.39

The data are presented as means ± s.d. (*N* = 6 individuals) and were analysed by two-way ANOVA (*P*<0.05) followed by Student–Newman–Keuls multiple comparison test. ^a,b^ Mean values not sharing a common letter are significantly different from each other (one way ANOVA, *P*<0.05).

**Table 7:** Fatty acid content of muscle expressed as mg/g of muscle (wet weight).

Fatty acids	VOC−		VOC+		*P* value		
	Fat	Lean	Fat	Lean	Diet	Line	Diet*Line
SFA	14.0±3.3	7.4±1.1	13.8±1.5	8.0±1.3	0.77	<10^−4^	0.64
MUFA	29.6±4.9	14.3±2.8	31.1±4.9	16.8±3.6	0.24	<10^−4^	0.77
n-6 PUFA	11.1±1.5	5.7±1.1	10.3±1.2	5.8±1.2	0.49	<10^−4^	0.41
20∶5 n-3	1.9±0.2	1.0±0.2	1.9±0.5	1.1±0.2	0.60	<10^−4^	0.62
22∶6 n-3	6.6±0.7	4.6±1.1	7.2±1.9	4.5±0.4	0.56	<10^−4^	0.50
n-3 PUFA	22.4±2.5	13.4±2.7	24.5±4.2	14.7±2.1	0.17	<10^−4^	0.76
n3/n6	2.03±0.11	2.33±0.14	2.38±0.15	2.57±0.19	10^−4^	5×10^−4^	0.39

The data are presented as means ± s.d. (*N* = 6 individuals) and were analysed by two-way ANOVA followed by Student–Newman–Keuls multiple comparison test. Differences were considered statistically significant at *P*<0.05

**Table 8:** Fatty acid profile of liver expressed as % of total fatty acids.

Fatty acids	VOC−		VOC+		*P* value		
	Fat	Lean	Fat	Lean	Diet	Line	Diet*Line
SFA							
14∶0	0.62±0.16	0.41±0.10	0.61±0.08	0.56±0.08	0.14	0.01	0.08
16∶0	16.39±1.54	14.31±2.22	14.83±1.89	15.15±1.70	0.64	0.25	0.12
17∶0	0.13±0.02	0.13±0.01	0.11±0.02	0.12±0.01	0.21	0.44	0.35
18∶0	6.67±0.92	6.63±0.31	6.54±0.84	6.52±0.47	0.67	0.91	0.96
20∶0	0.15±0.01	0.19±0.03	0.16±0.04	0.17±0.02	0.51	0.04	0.17
∑SFA	24.07±1.22	21.76±2.27	22.33±1.54	22.63±1.51	0.52	0.15	0.07
MUFA							
16∶1	0.82±0.17	0.60±0.15	0.80±0.28	0.74±0.13	0.47	0.08	0.31
18∶1	14.36±1.50	13.17±0.66	14.25±2.10	14.19±1.18	0.45	0.31	0.36
20∶1	1.78±0.80	1.68±0.45	1.79±0.66	1.74±0.35	0.89	0.76	0.91
22∶1	0.56±0.32	0.60±0.18	0.70±0.18	0.77±0.63	0.31	0.73	0.93
∑MUFA	17.52±2.15	16.04±0.78	17.54±2.23	17.43±1.27	0.32	0.27	0.34
n-6 PUFA							
18∶2 n-6	5.53±0.84	5.57±0.27	4.90±0.74	5.04±0.55	0.03	0.74	0.87
18∶3 n-6	0.21±0.09	0.23±0.04	0.13±0.03	0.16±0.04	0.003	0.19	0.81
20∶2 n-6	1.03±0.54	0.97±0.32	0.87±0.29	0.89±0.21	0.41	0.88	0.76
20∶3 n-6	1.17±0.24	1.31±0.30	1.28±0.36	1.00±0.16	0.39	0.55	0.08
20∶4 n-6	2.25±0.19	2.58±0.54	1.85±0.16	2.49±0.29	0.08	0.001	0.26
22∶2 n-6	0.08±0.05	0.09±0.03	0.10±0.03	0.10±0.02	0.20	0.86	0.52
∑n-6 PUFA	10.26±0.46	10.74±0.26	9.14±0.77	9.67±0.27	<10^−4^	0.02	0.89
n-3 PUFA							
18∶3 n-3	4.00±0.61	3.85±0.53	4.56±0.87	4.47±0.85	0.06	0.68	0.93
18∶4 n-3	0.93±0.69	0.78±0.24	0.74±0.31	0.80±0.33	0.61	0.78	0.56
20∶3 n-3	0.88±0.41	0.82±0.26	0.92±0.23	0.85±0.22	0.74	0.58	0.93
20∶4 n-3	0.89±0.35^ab^	1.0±0.46^ab^	1.41±0.47^a^	0.72±0.15^b^	0.45	0.08	0.02
20∶5 n-3	4.78±1.11	4.95±0.81	3.83±0.53	4.86±0.75	0.13	0.08	0.20
22∶5 n-3	0.87±0.46	1.14±0.19	1.31±0.36	1.46±0.38	0.02	0.16	0.68
22∶6 n-3	28.88±1.68	33.65±3.35	32.52±4.11	32.07±2.71	0.42	0.10	0.05
∑n-3 PUFA	41.2±2.4^b^	46.2±3.36^a^	45.3±3.3^ab^	45.2±2.5^a^	0.20	<0.05	0.04
Ratios							
SFA/PUFA	0.47±0.04^a^	0.38±0.06^b^	0.4±0.04^ab^	0.4±0.04^ab^	0.48	<0.05	0.04
n3/n6	4.02±0.26	4.30±0.30	5.01±0.78	4.68±0.33	0.002	0.89	0.13

The data are presented as means ± s.d. (*N* = 6 individuals) and were analysed by two-way ANOVA (*P*<0.05) followed by Student–Newman–Keuls multiple comparison test. ^a,b^ Mean values not sharing a common letter are significantly different from each other (one way ANOVA, *P*<0.05).

## Discussion

In the present study, we investigated the molecular and phenotypic response of two rainbow trout lines (fat and lean) to a vegetable oil based diet either with or without gelatinized starch.

### Effect of dietary carbohydrates in fish fed with vegetable oils

The absolute difference in carbohydrate intake (1.8% *vs.* 17.1%) between the two dietary groups as compared to protein intake (always above the requirement) enables us to consider that majority of the effects are linked to carbohydrate intake. When included at an acceptable level (20%) in the diet, carbohydrate (gelatinised starch) had no adverse impact on feed intake and growth. However, feeding of dietary carbohydrate caused a relatively moderate postprandial hyperglycemia that lacked temporal induction following a single meal, probably due to long term adaptation. These findings corroborate our previous observations in the two trout lines [23]. Likewise, the intake of dietary carbohydrate resulted in a proportionately heavier liver and viscera, which can be attributed to increased glycogen and fat deposition, respectively [29]. The phenotypic observations thus suggest the influence of dietary carbohydrate on the metabolism of the fish. Endorsing this, molecular analysis revealed a well-known mammalian response in hepatic glucose metabolism that includes strong induction of glycolytic GK transcripts and reciprocal reduction of gluconeogenic G6Pase1 transcripts, the latter being unusual in trout [30]–[32]. Specifically in these lines, the first isoform of G6Pase is known to respond to nutritional (refeeding) regulation [22]. There were also a few ambiguities related to dietary starch intake such as the contrasting response of intestinal glucose transporters (inert SGLT1 and enhanced GLUT2) that cannot validate an improvement in glucose uptake [33]. Moreover, the unaltered HK and GLUT4 expression in the muscle and adipose tissue reiterates the recognized poor ability of the peripheral tissues to adapt to high influx of glucose through the diet [34]–[36]. Therefore in brief, this study confirms the significant influence of dietary carbohydrate intake on glucose metabolism in the liver but not in the peripheral tissues, as evidenced before [23].

Consumption of a diet rich in carbohydrates is known to stimulate the lipogenic pathway through transcriptional mechanisms linked to enhanced glucose metabolism. This flux depends also on the availability of cofactors such as NADPH produced by the pentose phosphate pathway and lipogenic substrates [17], [37]. Our previous study in these two lines clearly demonstrated the existence of the above phenomenon through an increase in the hepatic transcript abundance of GK followed by enhanced mRNA levels and activity of G6PD (involved in the production of NADPH), ACLY transcripts (involved in the transition of glycolytic carbon to lipogenic substrates) and FAS activity (key enzyme of *de novo* lipogenesis) [23]. However in the present study, despite the huge induction of GK, dietary influence was conspicuously absent in all of the above mentioned lipogenic enzyme transcripts. The main causal differences can be dietary changes related to fish oil replacement [8] or physiological changes related to the larger size of the fish [38]. The only notable exception was carbohydrate intake enhanced ACC expression, which might have prevailed due to the presence of glucose response element in their promoter region as seen in mammals [37]. The unaltered plasma triglyceride levels of the carbohydrate fed group seemed to be related to the lipogenic profile rather than the potentially elevated intestinal lipid uptake, which is indicated by the transcript abundance of key chylomicron assembly proteins (MTP, ApoA1 and ApoA4). On the contrary, we found a paradoxical increase in the plasma cholesterol levels after carbohydrate intake. Considering the isolipidic nature of the two diets and neutral triglyceride levels, it is difficult to interpret this atypical link between carbohydrate intake, chylomicron synthesis and cholesterol metabolism [39]. Another contradiction related to fatty acid oxidation is the enhanced expression of hepatic CPT1b following carbohydrate intake. Normally in higher vertebrates, fatty acid oxidation and glucose oxidation/lipogenesis are known to be reciprocally regulated, because the provision of glucose inhibits fatty acid oxidation [40]. Further, the parallel higher expression of ACC and CPT1b are unusual even if at the transcriptional level, considering the fact that malonyl-CoA (product of ACC) is an inhibitor of CPT1. But, such paradoxical imbalance in the regulation of lipogenesis and lipid oxidation pathway has been previously reported in trout after insulin infusion [41]. Overall our results in hepatic lipid metabolism disagree with well known effects of carbohydrate intake, suggesting a disturbance characterized by unexplained shift from fatty acid storage to oxidation. In the peripheral tissues, dietary manipulation yet again had no influence on lipid metabolism, reasserting their unresponsiveness to carbohydrate intake [23].

Fatty acid desaturases and elongases are key enzymes of the fatty acid bioconversion pathway, which can influence whole body lipid composition. Dietary carbohydrate intake enhanced the transcription of key desaturase (D6D) and elongase (Elovl5 and Elovl2) enzymes involved in LC-PUFA synthesis, in both these lines, in agreement with few other mammalian and fish studies [10], [42]–[44]. The influence of carbohydrates on these enzymes can be effected through certain critical nodes such as an increased production of essential reducing power in the form of NADPH related to G6PD [45], [46]; availability of substrate for elongase in the form of malonyl-CoA related to ACC [47]; insulin action mediating the effect of glucose through transcriptional factors and presence of glucose response elements in the promoter regions of the encoding genes [42], [48], [49]. Out of which, we observed an increase in the hepatic expression of ACC and PPARγ associated to carbohydrate intake. PPARs are a family of nuclear receptors that regulates lipid and glucose metabolism, allowing adaptation to the prevailing nutritional environment. Especially, PPAR activation is known to promote glucose utilization and D6D gene is known to contain a PPAR response element [50]. In mice, the activation of PPARγ reportedly increases glycolysis, fat storage, fatty acid desaturation, elongation and restore insulin sensitivity [51]. For the first time, we provide evidence that piscine PPARγ acts similar to their mammalian counterpart, thereby ruling out previous speculations of functional divergence [52]. Other transcriptional factors such as SREBP-1 and PPARα that play a major role in the regulation of LC-PUFA synthesis [53], [54] were not influenced by carbohydrate intake. As we observed, nutritional regulation of fatty acid bioconversion enzymes independent of changes in SREBP-1 and PPARα have been previously reported in rats [55]. Based on our results, PPARγ seems to be the key transcription factor that mediates the carbohydrate induced response of desaturase and elongase enzymes in the liver, but not in the intestine, suggesting tissue specific regulation [56]. On the other hand, it is important to note that previously when the two lines were fed carbohydrates as part of fish oil based diet, transcriptional stimulation of D6D, Elovl5 and Elovl2 were absent probably as a result of higher LC-PUFA content and associated feedback inhibition [23], [24]. Therefore, the induction that we find now is in fact an amplified response due to the presence of carbohydrate in conjunction with the reduced content of n-3 LC-PUFA in the diet, which indeed validates our hypothesis at the transcriptional level. Though the exact mechanisms are unknown, changes in dietary fatty acid composition (chain length and desaturation) and subsequently altered cellular metabolism as a result of energy substrate crosstalk is the most likely explanation.

Concerning fatty acid phenotype, we found no increase in the n-3 LC-PUFA content of the flesh corresponding to the molecular augmentation of desaturase/elongase expression in the liver and intestine. Instead, they closely resembled the fatty acid profile of the respective diet consumed, as commonly observed [4]. It is well known that the fatty acid composition of an animal depends not only on desaturation/elongation, but also on other interacting aspects of lipid metabolism such as b-oxidation, substrate availability, tissue uptake and hormonal status [4], [42], [57]. High levels of oxidation of C_18_ PUFA, which subsequently allows only small proportions for bioconversion, can be one possible explanation [58]. The elevated CPT1b expression in the carbohydrate fed group coincides with this hypothesis. The uptake and selective incorporation of fatty acids in the cellular fractions of the muscle can also have significance in the final lipid composition [59], [60]. The expression of muscle lipid uptake markers, namely LPL, VLDLR and CD36 suggests no diet induced differences in fatty acid uptake. Besides, the impact of post-transcriptional modifications and related changes in enzyme activity on the final fatty acid profile is not known, as most of our data represent only the transcriptional changes. Eventually, the hypothesized additive influence of dietary carbohydrate and low n-3 LC-PUFA content (vegetable oil) on PUFA biosynthesis was evident at the molecular level, but did not elaborate into a beneficial fatty acid phenotype.

### Effect of fat and lean genotypes in fish fed with vegetable oils

The superior growth performance of the L line as compared to the F line was known to be an outcome of improved feed efficiency and protein utilization, whereas the voluntary feed intake used to be the same under a fish oil based dietary regime [21], [23], [61]. But with the switch to vegetable oil based diet in the present study, the L line consumed slightly more feed than the F line irrespective of the carbohydrate content and thereby exhibited more pronounced increase in body weight. Such genotype specific preferential acceptance of vegetable oil diet adds to the basic understanding that trout can discriminate between feeds with different oil sources [62].

Concerning glucose metabolism, the F line showed a marginal but significantly lower postprandial plasma glucose levels (8 h) than the L line, following a high carbohydrate meal. There are two possible reasons, one is enhanced storage of excess glucose as glycogen as suggested by the higher hepato-somatic index of the F line fish and the other is increased glucose uptake in the white muscle of F line as evidenced by GLUT4 transcript abundance. These observations corroborate the better glycemic control and improved muscle glycolysis in the F line, reported under standard (10%) carbohydrate regime [21], [22]. However in our preceding study with high level of carbohydrates given as part of fish oil based diet, contradictorily, the F line did not exhibit an improved glycemic regulation despite having a higher ability to store excess glucose in the liver as glycogen or fat. Weaker control over hepatic endogenous glucose production in the F line was suggested to outdo its higher glycolytic ability [23]. This genotypic difference in glycolysis and gluconeogenesis disappeared under the vegetable oil based dietary regime in the present study, but it is hard to give substantial explanation for cause or consequence.

In previous studies, transcriptional analysis (after feeding fish oil based diets with or without carbohydrates to the two trout lines) demonstrated the higher potential of the F line in intestinal lipid uptake, hepatic *de novo* fatty acid synthesis (further enhanced by dietary carbohydrates) and fatty acid bioconversion in both liver and intestine [23], [24]. We hypothesized that replacing fish oil with vegetable oil may augment this genetic potential of the F line because vegetable oil lacks n-3 LC-PUFA, the critical component in fish oil responsible for down regulating the genes encoding enzymes involved in lipogenesis and fatty acid bioconversion [54], [57], [63]. But contrary to our expectation, changing the dietary lipid source eliminated the genetic advantage of the F line in chylomicron synthesis, lipogenesis as well as fatty acid desaturation and elongation. The only exception was the consistently higher hepatic expression of the pentose pathway enzyme G6PD in the F line, irrespective of changes in dietary composition across studies [22], [23], [64]. So this enzyme connecting glucose and lipid metabolism may be a key marker of the fat muscle genotype.

On the other hand, we noticed few reversals in genotypic differences in the presence of dietary vegetable oil, the most prominent being the circulating triacylglycerol (TAG) phenotype. The previously reported higher plasma TAG linked to enhanced lipogenic ability in the F line [23] was inversely lowered in the F line, together with the annulation of genotypic difference in lipogenesis. A study in Atlantic salmon suggested that the differences between families in plasma TAG levels were influenced by peripheral tissue uptake rather than hepatic lipid metabolism, relating it to their lipid storage phenotype [65]. Correspondingly in the present study, the lower plasma TAG of the F line could be associated to the higher uptake of lipids in the muscle (adipocytes) as indicated by the transcript abundance of VLDLR, a receptor mediating internalisation and clearance of lipoproteins and CD36, a fatty acid translocase that determines long chain fatty acid uptake and lipid metabolism. The increased level of transcripts encoding the VLDLR and CD36 in the F line has already been reported, identifying them (and not LPL) as relevant molecular markers for fat deposition and circulating lipid uptake in the white muscle of rainbow trout [66]. A second reversal was evident in the transcriptional factor SREBP1c, which was higher in the liver and adipose tissue of the L line after fish oil replacement [23]. But, this activation was not accompanied by downstream changes in the lipogenic enzymes. Key factors regulating multiple facets of hepatic lipid metabolism such as SREBP1c, PPARα and PPARγ was uniformly up-regulated in the L line. But in target response, except for a higher expression of hepatic CPT1b in the L line related to PPARα activation, no other correlation was noticeable.

Recent data suggest that n-3 LC-PUFA content of the flesh is a highly heritable trait in salmonids [18]. Fatty acid deposition and the activity of the bioconversion pathway are also known to be dependent on the genetic background of the fish [5], [67], [68]. However, changes in dietary lipid source (i.e., vegetable oil) can alter the genetic potential; the magnitude and direction of response varies between family groups [19], [56]. In accordance, we found that the inherently higher fatty acid bioconversion ability of the F line was eliminated or reversed (intestinal D6D) after replacing fish oil with vegetable oil [23], [24], [64]. But the key enzymes (desaturase and elongases) and transcriptional factors (PPARs and SREBP1c) were not coordinately regulated in both lines to portray a defined pattern of expression with biological significance. At the phenotypic level, dietary vegetable oil did not affect the higher muscle lipid content (selection criterion) of the F line and thus it retained greater quantity of saturated and unsaturated fatty acids in absolute terms, unlike in salmon where differences in muscle lipid content between the fat and lean genotype reportedly diminished in 100% vegetable oil fed group [5]. Nevertheless in relative terms, the n-3 LC-PUFA content of the F line flesh was slightly lower than the L line. This suggests that the L line may have higher responsiveness to low dietary n-3 LC-PUFA, up-regulating the biosynthetic pathway when fed diets with vegetable oil. Overall, our results emphasize that the vegetable oil based dietary regime alters lipid metabolism depending on the genetic background and the LC-PUFA biosynthesis pathway showed no pertinent genotypic difference when assessed by gene expression and fatty acid composition.

## Conclusion

This study demonstrates that carbohydrate intake when coupled with lower dietary content of n-3 LC-PUFA (vegetable oil) promotes the inherent LC-PUFA biosynthetic pathway, regardless of the genetic background of the fish. At the molecular level, this was confirmed by the enhanced transcriptional response of key desaturase and elongase enzymes, mediated through the PPARγ regulatory factor in the liver of both lines; however, the final fatty acid profile of the flesh did not evidence a correlative augmentation of LC-PUFA content. Moreover, the documented genetic pre-disposition of higher fatty acid bioconversion in the fat genotype disappeared under the vegetable oil based diet regime. Dietary macro-nutrient interface is thus a critical aspect to be deliberated during the progressive shift towards plant based feeds and while analyzing nutrient × genotype interactions.
